# Epithelial-Mesenchymal Transition in Colorectal Carcinoma: Comparison Between Primary Tumor, Lymph Node and Liver Metastases

**DOI:** 10.3389/fonc.2021.662806

**Published:** 2021-05-11

**Authors:** Ana Pavlič, Kristian Urh, Katarina Štajer, Emanuela Boštjančič, Nina Zidar

**Affiliations:** Institute of Pathology, Faculty of Medicine, University of Ljubljana, Ljubljana, Slovenia

**Keywords:** epithelial-mesenchymal transition, mesenchymal-epithelial transition, colon cancer, metastases, miRNA

## Abstract

There is emerging evidence suggesting that epithelial-mesenchymal transition (EMT) and mesenchymal-epithelial transition (MET) play an important role in colorectal carcinoma (CRC), but their exact role remains controversial. Our aim was to analyze the miR-200 family as EMT markers and their target genes expression at invasive tumor front and in nodal and liver metastases. Sixty-three formalin-fixed paraffin-embedded tissue samples from 19 patients with CRC were included. Using a micropuncture technique, tissue was obtained from central part and invasive front of the primary tumor, and nodal and liver metastases. Expression of the *miR-200* family and their target genes *CDKN1B, ONECUT2, PTPN13, RND3, SOX2, TGFB2* and *ZEB2* was analyzed using real-time PCR. We found *miR-200* family down-regulation at invasive front compared to central part, and up-regulation of *miRNA-200a/b/c* and *miR-429* in metastases compared to invasive front. At invasive front, *TGFB2* was the only gene with inverse expression to the miR-200 family, whereas in metastases inverse expression was found for *ONECUT2* and *SOX2. CDKN1B, PTPN13* and *ZEB2* were down-regulated at invasive front and up-regulated in metastases. Our results suggest the involvement of partial EMT at invasive tumor front, and partial MET in metastases in CRC, based on miR-200 family and its target genes expression.

## Introduction

Significant progress has been made in recent decades in our understanding of cancer development, progression and metastasizing. Among pathogenetic mechanisms of cancerogenesis, epithelial-mesenchymal transition (EMT) has gained much attention. There is emerging evidence suggesting that EMT plays an important role in human cancer, but its role remains controversial (1–3).

EMT is a fundamental process during embryogenesis and is later physiologically silenced. In adult life, EMT can be reactivated in various conditions, such as wound healing, fibrosis and cancer (4, 5). During EMT, epithelial cells change from stationary polarized cells to mobile spindle cells. In cancer, this transition is assumed to be the basis for progression and metastasis, enabling cells to migrate from the primary tumor (6, 7). Once disseminated to a distant site, cancer cells are believed to regain epithelial properties in a reverse process, referred to as mesenchymal-epithelial transition (MET) (8, 9). Complete EMT includes a switch in intermediate filament expression, an alteration in intercellular junction composition and a concurrent change of cell morphology (10, 11). It appears that full EMT rarely occurs in human cancer though it does exist, e.g., in spindle cell carcinoma, which can be regarded as a “positive” control when studying the markers of EMT (12). Markers of EMT include classical and desmosomal cadherins, intermediate filaments and transcription factors, but their expression patterns can be variable in diseases in which EMT presumably plays an important role (13, 14).

In the majority of human cancer types, however, the morphologic criterion of a complete shift from an epithelial to a spindle phenotype during EMT is seldom met. Instead, cancer progression for the majority of cancers might only depend on partial EMT (15, 16). Cells undergoing partial EMT are difficult to identify due to their phenotypic heterogeneity, the transient and reversible nature of EMT and variable expression of EMT markers (15). Evidence of partial EMT often relies on activation of EMT transcription factors and their regulatory molecules, such as microRNAs (3, 17, 18). Previous studies suggest that *miR-200* family is among the most sensitive markers of EMT and can be used to study full and partial EMT (12, 19–23).

A few studies have suggested that partial MET might occur at the metastatic site (24, 25) but the dynamics of EMT and MET in cancerogenesis is still poorly understood. The aim of this study was to analyze the expression of the *miR-200* family and their target genes as markers of EMT in locations crucial for the progression of colorectal carcinoma (CRC), i.e., the central part of the tumor and invasive tumor front, as well as lymph node and liver metastases. The dynamics of EMT markers expression profiles might provide insight into the possible role of EMT/MET in cancer progression and metastasis formation.

## Materials and methods

### Patients and Tissue Samples

This retrospective study was approved by the National Medical Ethics Committee, Republic of Slovenia, Ministry of Health (reference number 0120-88/2020/3) and carried out following the rules of the Declaration of Helsinki. All authors had access to the study data and had reviewed and approved the final manuscript.

The study included patients with CRC who had been treated surgically, including resection of the primary tumor and regional lymph nodes, as well as liver metastases if present. Patients were divided into three groups: patients with CRC and lymph node metastases (N+ M0), patients with CRC and liver metastases but without lymph node metastases (N0 M+) and patients with CRC and both lymph node and liver metastases (N+ M+). Patients with neoadjuvant radiochemotherapy were excluded from the study. None of the patients had microsatellite unstable cancer.

After surgery, resection specimens were dealt with according to standard procedures. After 24-hour fixation in formalin, representative samples from the tumor, all lymph nodes and resection margins were taken, 3-4 µm-thick sections were cut and stained with eosin and hematoxylin. pTNM stage was determined after the primary resection (26).

For the purposes of this study, all slides were re-examined. Tumor budding was determined according to recommendations as Bd1-Bd3 (Bd1 0-4 buds/0.785 mm^2^, Bd2 5-9 buds/0.785 mm^2^, Bd3 ≥10 buds/0.785 mm^2^) (27). Poorly differentiated (PDC) were analyzed and graded as G1-G3 (G1: <5; G2: 5-9; G3: ≥ 10 PDC in a 20x objective lens field) (28, 29). Representative slides of the tumor and metastases were selected and corresponding paraffin blocks were retrieved from the archives of the Institute of Pathology, Faculty of Medicine, University of Ljubljana. Tissue was obtained from four locations using micropuncture technique, using a 0.6 mm needle: central part of the primary tumor, invasive tumor front and lymph node and liver metastases if present.

### RNA Isolation

#### RNA Isolation From Formalin-Fixed Paraffin Embedded Tissue Samples

For the isolation procedure, 3 punches were made at each location. Total RNA isolation was performed using a MagMax FFPE DNA/RNA Ultra kit (Applied Biosystems, Foster City, CA, USA) according to the manufacturer’s protocol with a modification. Protease digestion was performed overnight at 56°C with shaking for 15 s at 300 rpm every 4 min. The concentration and quality of the isolates were assessed with a spectrophotometer ND-1000 or ND-One (Nanodrop, Thermo Fisher Scientific, Waltham, MA, USA) at the wavelengths 260, 280 and 230 nm.

#### RNA Quality Assessment

As quality control, reverse transcription (RT) of *RNU6B*, a housekeeping small nuclear RNA gene, was used followed by amplification using quantitative real-time PCR (qPCR) and TaqMan methodology (Thermo Fisher Scientific, Waltham, MA, USA). All of the samples included in the study had passed this initial quality control and those that did not amplify were not included in the study. Positive and negative amplification of *RNU6B* was in 100% correlation with positive and negative amplification of *GAPDH* (100 bp) used as initially quality control in previous research (data not shown) (19, 30). For selected genes, we chose TaqMan primers and probes that amplify and detect PCR products less than 100 bp long (**Table 1**).

**Table 1:** Probes used for miRNAs and mRNAs quantification using quantitative real-time PCR (qPCR).

Probe Name	Probe ID Number	Length of PCR Product (bp)
*B2M*	Hs 99999907_m1	75
*CDKN1B*	Hs00153277_m1	71
*IPO8*	Hs 00183533_m1	71
*ONECUT2*	Hs00191477_m1	57
*PTPN13*	Hs01106214_m1	65
*RND3*	Hs01003594_m1	91
*SOX2*	Hs04234836_s1	86
*TGFB2*	Hs01555416_m1	67
*ZEB2*	Hs01095318_m1	58
*RNU6B*	ID 001093	nd
*miR-141*	ID 000463	nd
*miR-200a*	ID 000502	nd
*miR-200b*	ID 002251	nd
*miR-200c*	ID 002300	nd
*miR-429*	ID 001024	nd
*miR-1274b*	ID 002884	nd

bp, base pair; nd, not defined.

### Efficiency Testing

A pre-designed mixture of probes and primers specific for miRNAs or target gene (mRNAs) expression was used. Prior to qPCR, three pools of RNA samples were created, obtained from primary tumor, lymph node and liver metastases. After RT, the cDNA of miRNAs and pre-amplified cDNA of mRNAs was diluted in four steps, ranging from 5-point dilution to 625-point dilution, and the probes were tested for qPCR efficiency. All the qPCR efficiency reactions were performed on a RotorGene Q (Qiagen, Hilden, Germany) in triplicate.

### Analysis of Expression Of *mir-200* Family

#### Reverse Transcription (RT)

Looped primers for specific reverse transcription (RT) of miRNAs and a MicroRNA TaqMan RT kit (Applied Biosystems, Foster City, CA, USA) were utilized following the manufacturer’s protocol. *RNU6B* and *miR-1247b* were used as reference genes (RGs). MicroRNAs, *miR-141*, *miR-200a*, *miR-200b*, *miR-200c* and *miR-429* were tested relative to the geometric mean of expression of *RNU6B* and *miR-1247b* (**Table 1**). Briefly, a 10 μL RT reaction master mix was performed with 10 ng of total RNA sample, 1.0 μL of MultiScribe Reverse Transcriptase (50 U/μL), 1.0 μL of Reverse Transcription Buffer (10×), 0.1 μL of dNTP (100 mM), 0.19 μL RNAase inhibitor (20 U/μL), and 2.0 μL of RT primer (5×). The reaction conditions were: 16°C for 30 min, 42°C for 30 min, 85°C for 5 min.

#### Quantitative Real-Time PCR (qPCR)

qPCR for miRNAs was carried out in a 10 μL PCR master mix containing 5.0 μL TaqMan 2× FastStart Essential DNA Probe Master (Roche, Basel, Switzerland), 0.5 μL TaqMan assay 20x, and 4.5 μL RT products diluted 100-fold. The qPCR reactions were performed on a RotorGene Q (Qiagen, Hilden, Germany) in duplicate, as follows: initial denaturation at 95°C for 10 min, 40 cycles for 15 s at 95°C (denaturation) and for 60 s at 60°C (primers annealing and elongation). The signal was collected at the endpoint of every cycle.

### Analysis of Expression of *miR-200* Family Target Genes

#### Reverse Transcription (RT)

Target mRNAs of the miR-200 family, *CDKN1B*, *ONECUT2*, *PTPN13*, *RND3*, *SOX2*, *TGFB2*, and *ZEB2* (**Table 1**), were analyzed relatively to the geometric mean of RGs, *IPO8* and *B2M*. mRNAs were reverse transcribed using a OneTaq RT-PCR Kit (New England Biolabs, Ipswich, MA, USA) using random primers according to the manufacturer’s instructions. Reverse transcription reactions were started with 3.0 µL (14-60 ng) of total RNA and 1.0 µL of Random Primer Mix incubated at 70°C for 5 min. The 10 μL RT master mix included 5.0 μL of M-MuLV Reaction Mix, 1.0 μL of M-MuLV reverse transcriptase and 4.0 μL of reaction mix after random priming. The reaction conditions were: 25°C for 5 min, 42°C for 60 min and 80°C for 4 min.

#### Pre-Amplification and Quantitative Real-Time PCR (qPCR)

Following RT, pre-amplification was performed using a TaqMan PreAmp Master Mix (Applied Biosystems, Foster City, CA, USA) in 10 µL according to the manufacturer’s protocol. The resulting PreAmp reaction was diluted 5-fold in all cases, except when investigating lymph node metastases, where it was diluted 25-fold. In the qPCR reaction 4.5 μL of the diluted sample was used in a 10 μL reaction volume with a 5.0 μL of 2x FastStart Essential DNA Probe Master Mix (Roche, Basel, Switzerland) and 0.5 μL of TaqMan 20X probe. Thermal conditions were applied as follows: 50°C for 2 min, initial denaturation at 95°C for 10 min and 40 cycles of denaturation at 95°C for 15 s and annealing at 60°C for 1 min. All qPCR analyses were performed on a Rotor Gene Q (Qiagen, Hilden, Germany) in duplicate. The signal was collected at the endpoint of each cycle.

#### Statistical Analysis of Experimental Data

The results were presented as relative gene expression. All Cqs were corrected for PCR efficiencies and the expression of the gene of interest (GOI, Cq_GOI_) was calculated relative to a geometric mean of RGs (Cq_RG_), named ΔCq. In CRC samples, mRNAs and miRNAs expression differences were compared between the central part of the tumor and the invasive front, invasive front and lymph node metastases and/or invasive front and liver metastases groups using ΔCq and the Wilcoxon Rank test. Lymph node and liver metastases groups were compared using ΔCq data and the Mann-Whitney U test. For all correlations/associations, Spearman rank-order correlation was used. Statistical analysis of data was performed using SPSS version 24 (SPSS Inc., Chicago, IL, USA). Differences were considered significant at p* *≤ 0.05.

## Results

### Patients and Tissue Samples

In total, we analyzed 63 tissue samples from 19 patients (13 males and 6 females) with CRC with lymph node and/or liver metastases. Nine patients had liver metastases at the time of the initial presentation and resection of the primary tumor, whereas 3 patients developed liver metastases later in the disease course and underwent additional surgical resection of the liver metastases (5, 11 and 44 months after resection of the primary tumor). pTNM stage was determined after the primary resection according to the international guidelines (26). We divided the patients in 3 groups, based on the presence of lymph node and/or liver metastasis: the N+ M0 group included 7 patients (mean age 76.0 ± 13.5 years; male:female 6:1), the N0 M+ group included 3 patients (mean age 72.0 ± 6.1 years, male:female 2:1) and the group N+ M+ group included 9 patients (mean age 69.3 years ± 16.5; male:female 5:4). Tumor budding was determined as grade Bd1, Bd2 and Bd3 in 9, 8 and 2 patients respectively. PDC were graded as G1, G2 and G3 in 6, 8 and 5 patients respectively. Demographic and clinicopathologic features and information about the analyzed tissue samples are presented in **Table 2**.

**Table 2:** Demographic and clinicopathologic features and information about the analyzed tissue samples.

Patient	Sex	Age (yrs)	Tumor location	pTNM*	Tumor budding	PDC	Analyzed tissue samples				Group
							Central part	Invasive front	Nodal metastases	Liver metastases	
1	M	54	Sigma	pT3N1	Bd2	G1	+	+	+	–	N+ M0
2	M	74	Sigma	pT3N1b	Bd2	G2	+	+	+	–	N+ M0
3	M	85	Sigma	pT3N1	Bd1	G1	+	+	+	–	N+ M0
4	M	62	Sigma	pT4aN2a	Bd2	G3	+	+	+	–	N+ M0
5	F	85	Ascendens	pT3N1a	Bd3	G3	+	+	+	–	N+ M0
6	M	91	Ascendens	pT3N1b	Bd1	G1	+	+	+	–	N+ M0
7	M	81	Ascendens	pT3N1b	Bd2	G2	+	+	+	–	N+ M0
8	F	79	Rectum	pT3N0M1a	Bd1	G2	+	+	–	+	N0 M+
9	M	69	Rectum	pT1NX	Bd2	G3	+	+	–	+	N0 M+
10	M	68	Rectosigma	pT3N0M1a	Bd1	G1	+	+	–	+	N0 M+
11	F	70	Sigma	T4N1M1a	Bd1	G1	+	+	+	+	N+ M+
12	M	31	Sigma	T3N2aM1a	Bd1	G2	+	+	+	+	N+ M+
13	M	66	Sigma	T4aN1bM1a	Bd2	G2	+	+	+	+	N+ M+
14	M	68	Sigma	T4aN2aM1a	Bd2	G3	+	+	+	+	N+ M+
15	M	68	Descendens	T3N1b	Bd1	G1	+	+	+	+	N+ M+
16	F	82	Descendens	T4aN1bM1a	Bd2	G2	+	+	+	+	N+ M+
17	F	68	Rectum	T4aN1a	Bd1	G2	+	+	NA	+	N+ M+
18	M	83	Ascendens	T4aN2aM1a	Bd3	G3	+	+	+	NA	N+ M+
19	F	88	Rectosigma	T4aN2bM1a	Bd1	G2	+	+	+	NA	N+ M+

+, present; 0, absent; NA, not available; PDC, poorly differentiated clusters *, pTNM as defined after resection of the primary tumor.

### Expression of the *miR-200* Family

**Figure 1:**
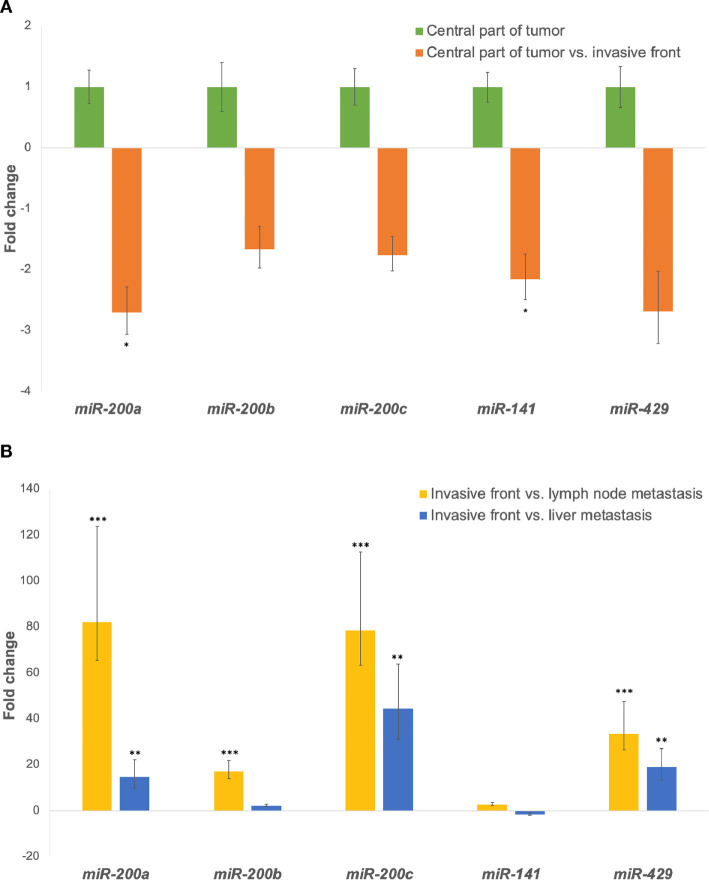
**(A)** Expression of the *miR-200* family at the invasive front in comparison to the central part of the tumor. *p ≤ 0.05. **(B)** Expression of the *miR-200* family in lymph node and/or liver metastases in comparison to the invasive tumor front. **p ≤ 0.01; ***p ≤ 0.001.

When comparing the invasive front to the central part of the tumor, all investigated miRNAs were down-regulated in all groups, as shown in **Figure 1A**.

All investigated miRNAs were up-regulated in lymph node metastases compared to the invasive front. Additionally, expression of all investigated miRNAs, except *miR-141*, were up-regulated in liver metastases when compared to the invasive front, as shown in **Figure 1B**. Fold changes and p-values for all investigated comparisons are presented in **Table 3**.

**Table 3:** Fold changes and p-values for all investigated comparisons for the *miR-200* family with Wilcoxon signed ranks test.

	Fold changes and p-values (2-tailed)				
	*miR-200a*	*miR-200b*	*miR-200c*	*miR-141*	*miR-429*
Central part vs. invasive front	-2.70 (0.011)	/	/	-2.15 (0.034)	/
Invasive front vs. lymph node metastasis	82.17 (< 0.001)	17.14 (< 0.001)	78.51 (< 0.001)	/	33.51 (0.001)
Invasive front vs. liver metastasis	14.72 (0.008)	/	44.5 (0.004)	/	19.01 (0.008)

**Figure 2:**
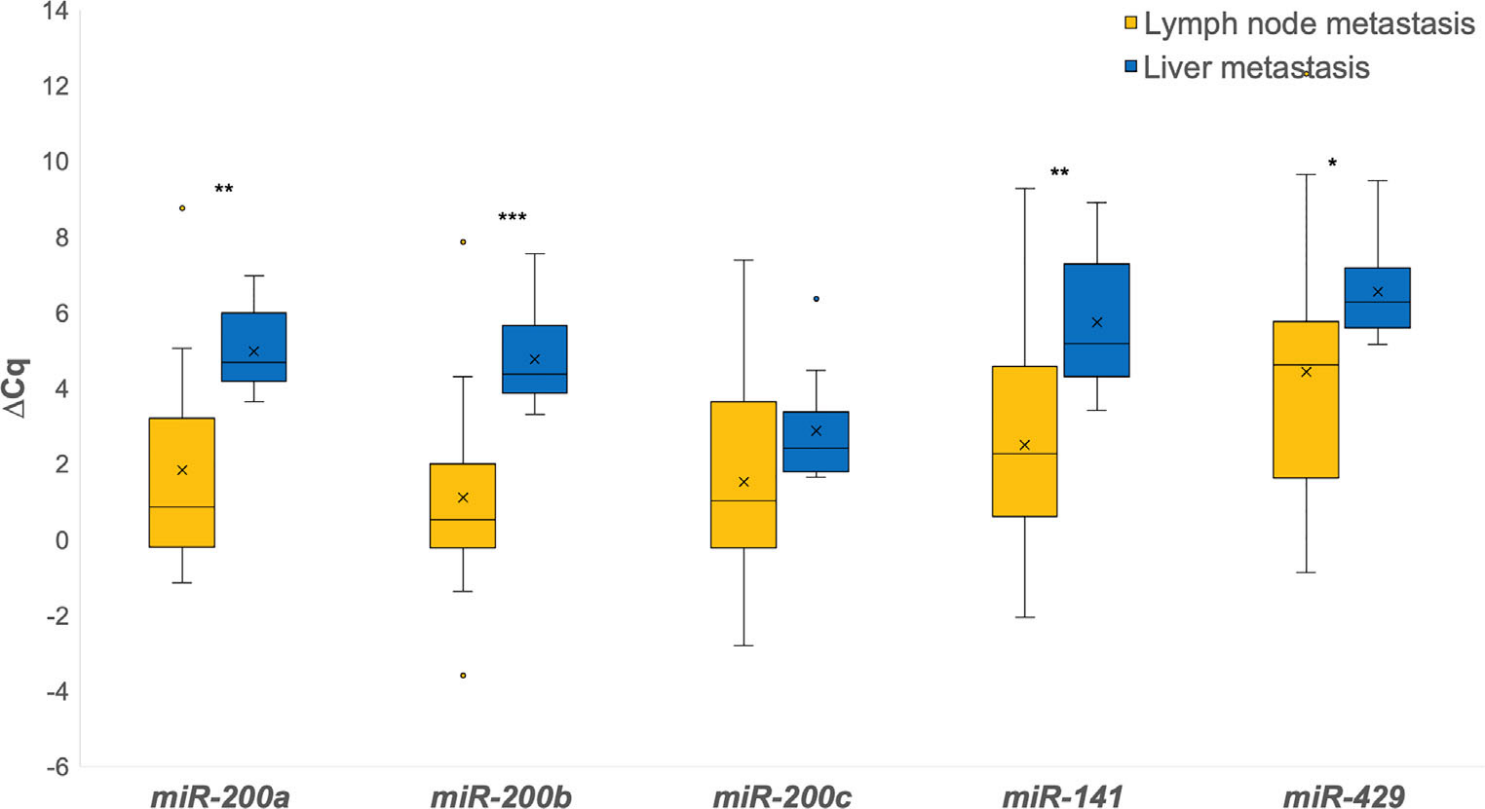
Expression of the *miR-200* family in lymph node metastases in comparison to liver metastases. *p ≤ 0.05; **p ≤ 0.01; ***p ≤ 0.001.

When investigating the differences between lymph node and liver metastases, all miRNAs were down-regulated in liver metastases when comparing them independently to lymph node metastases. Statistically significant differences were found for *miR-200a* (p = 0.003), *miR-200b* (p = 0.001), *miR-141* (p = 0.005) and *miR-429* (p = 0.016), as shown in **Figure 2**.

### Expression of the Target Genes of the *miR-200* Family

**Figure 3:**
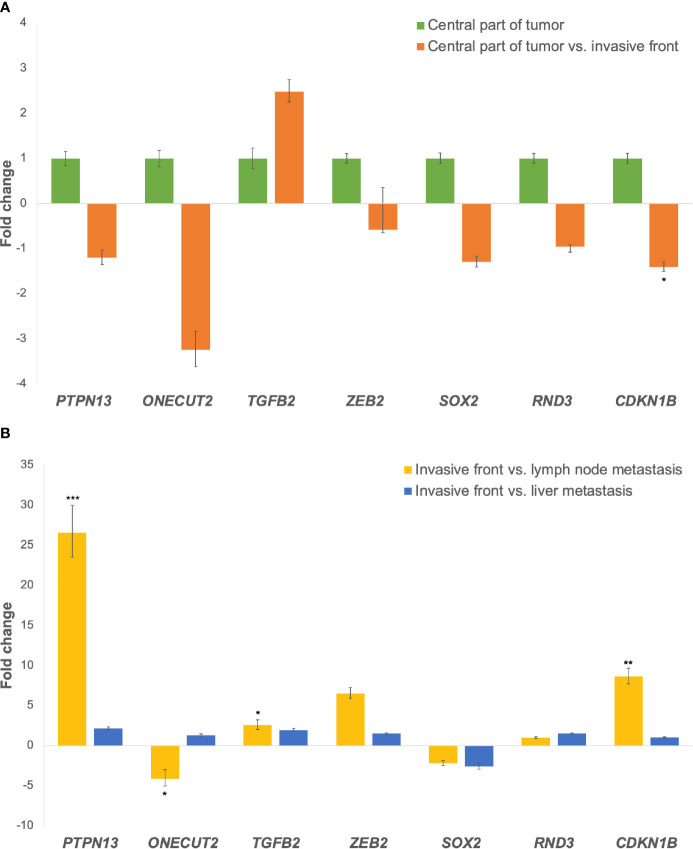
**(A)** Expression of the *miR-200* family target genes at the invasive tumor front in comparison to the central part of the tumor. Legend: *p ≤ 0.05. **(B)** Expression of the *miR-200* family target genes in lymph node and liver metastases in comparison to the invasive tumor front. Legend: *p ≤ 0.05; **p ≤ 0.01; ***p ≤ 0.001.

Expression of all investigated genes between the central part of the tumor and the invasive front as fold changes is shown in **Figure 3A**. All investigated genes in this comparison were down-regulated, except *TGFB2*, which was up-regulated.

In lymph node metastases compared to the invasive front of the tumor, *ONECUT2* and *SOX2* were down-regulated, while *PTPN13*, *TGFB2*, *ZEB2*, *RND3* and *CDKN1B* were up-regulated. When comparing liver metastases to the invasive tumor front, *SOX2* was down-regulated, while *PTPN13*, *ONECUT2*, *TGFB2*, *ZEB2*, *RND3* and *CDKN1B* were up-regulated, as shown in **Figure 3B**. Fold changes and p-values for all investigated comparisons among groups are shown in **Table 4**.

**Table 4:** Expression of the *miR-200* family related genes (Wilcoxon signed ranks test).

	Fold changes and p-values (2-tailed)						
	*PTPN13*	*ONECUT2*	*TGFB2*	*ZEB2*	*SOX2*	*RND3*	*CDKN1B*
Central part vs. invasive front	/	/	/	/	/	/	-1.41 (0.045)
Invasive front vs. lymph node metastasis	26.58 (0.001)	-4.15 (0.031)	2.56 (0.016)	/	/	/	8.64 (0.002)
Invasive front vs. liver metastasis	/	/	/	/	/	/	/

**Figure 4:**
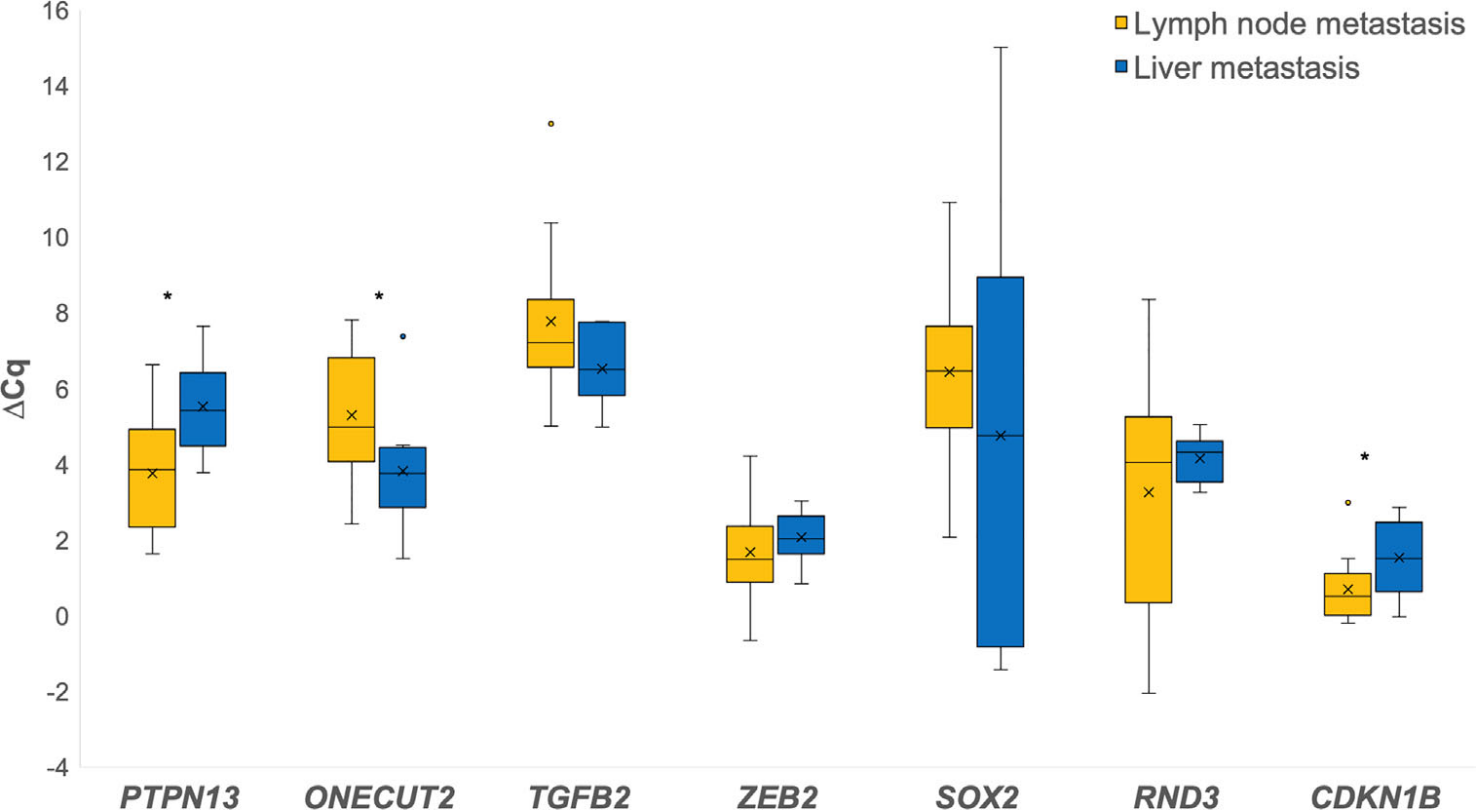
Expression of the investigated genes in lymph node metastases in comparison to liver metastases. *p ≤ 0.05.

When comparing liver metastases to lymph node metastases for the investigated genes, *PTPN13* (p = 0.012), *ZEB2*, *RND3*, *CDKN1B* (p = 0.036) were down-regulated, while *ONECUT2* (p = 0.046), *TGFB2*, *SOX2* were up-regulated in the liver metastases as shown in **Figure 4**.

### Correlations Between *miR-200* family and Their Target Genes

Spearman coefficient of correlation revealed moderate or weak positive correlation between *miR-200a* and *PTPN13*, *ZEB2*, and *CDKN1B*, respectively. *miR-200b* was in weak positive correlation with *PTPN13* and *ZEB2*. *miR-200c* was in weak positive correlation with *PTPN13*, *ZEB2* and *CDKN1B*. *miR-141* did not correlate significantly with any of the investigated genes, while *miR-429* was in weak positive correlation with *ZEB2*. Results are summarized in **Table 5**.

**Table 5:** Comparison between the *miR-200* family and investigated genes expression (Spearman correlation coefficients and corresponding p-values in brackets).

	*PTPN13*	*ONECUT2*	*TGFB2*	*ZEB2*	*SOX2*	*RND3*	*CDKN1B*
***miR-200a***	0.444 (0.001)	/	/	0.462 (0.000)	/	/	0.330 (0.010)
***miR-200b***	0.343 (0.012)	/	/	0.312 (0.019)	/	/	/
***miR-200c***	0.399 (0.003)	/	0.319 (0.033)	0.377 (0.004)	/	/	0.264 (0.045)
***miR-141***	/	/	/	/	/	/	/
***miR-429***	/	/	/	0.274 (0.037)	/	/	/

## Discussion

We analyzed the expression of the *miR-200* family, known to be the master regulator of EMT, and their target genes, to investigate the role of EMT in CRC invasion and metastasizing. We obtained tumor tissue from locations essential for tumor growth and progression, i.e., the invasive tumor front and lymph node and/or liver metastases. The results of our study suggest the involvement of partial EMT at the invasive tumor front and the involvement of partial MET in both lymph node and liver metastases. This is based on the expression patterns of the *miR-200* family in these critical locations, showing *miR-200* family down-regulation at the invasive front compared to the central part of the tumor, suggesting the involvement of EMT in invasive tumor growth, and up-regulation in both lymph node and liver metastases compared to the invasive front, suggesting the involvement of MET in metastasis formation.

Similar to previous studies, we did not observe any morphologic evidence of full EMT, i.e., transition from an epithelioid to a spindle phenotype of carcinoma cells at the invasive front. Furthermore, no morphologic evidence of transition from a spindle to an epithelioid phenotype was noted in the metastases. These findings support the hypothesis that EMT and MET do play an important role in CRC growth and metastases, not as full EMT/MET but only as partial EMT/MET.

When we compared the invasive front to the central part of the tumor, all five investigated miRNAs (*miR-200a/b/c, miR-141, miR-429*) were down-regulated in all samples, the differences being significant for *miR-200a* and *miR-141*. Several previous studies have suggested that impaired *miR-200* expression may lead to EMT initiation and eventually to cancer dissemination. Decreased expression of *miR-200* has previously been reported at the invasive front of metastatic CRC (24, 25, 31–33). Specifically, Paterson et al. found *miR-200a/b/c* to be down-regulated at the invasive front with degraded basement membrane (25) and Knudsen et al. demonstrated *miR-200b* down-regulation in the tumor budding cells of CRC (31). Muto et al. also showed a statistically significant difference in *miR-200c* expression between the invasive front of metastatic and non-metastatic tumors (32), whereas no significant difference was observed by Jepsen et al. (33). It is believed that down-regulation of *miR-200* and subsequent up-regulation of their target genes promotes a switch in cell phenotype, resulting in an increased invasiveness and metastatic potential of tumor cells.

Furthermore, we found strong up-regulation of *miRNA-200a/b/c* and *miR-429* in both lymph node and liver metastases in comparison to the invasive tumor front. All differences were statistically significant except for *miR-200b* in liver metastases. Only a few previous studies have focused on the expression of EMT markers in locations critical for carcinoma progression and metastasizing. Similar to our study, Hur et al. used the miR-200 family as markers of EMT and demonstrated down-regulation of *miR-200c* at the CRC invasive front and up-regulation in matched liver metastases (24). They concluded that *miR-200c* probably plays a pivotal role in CRC metastases. Our results support their findings, since we also showed significant up-regulation of *miR-200c* in matched lymph node and liver metastases. In contrast, Li et al. demonstrated down-regulation of *miR-200c* in matched CRC liver metastases (34). Previous studies using *miR-200a* on matched metastases in CRC are very scarce. Cristobal et al. observed no significant difference in the expression of *miR-200a* between primary CRC and matched liver metastases (35). Interestingly, we found *miR-200a* to be a reliable marker, showing both significant down-regulation at the invasive front and up-regulation in the metastases. Paterson et al. immunostained tumors and local lymph node metastases with miR-200b and demonstrated weak staining of *miR-200b* at the invasive front and intense staining in metastases and vascular carcinoma deposits. Their results provide some evidence of cells undergoing EMT at the invasive front and recapitulating the phenotype of the primary tumor at metastatic sites (25).

To the best of our knowledge, this is the first study to compare the expression of the *miR-200* family in CRC with matched lymph node and liver metastases. Our results suggest that partial EMT/MET is involved in metastasis formation regardless of hematogenous or lymphogenic tumor spread. Except for *miR-141*, all tested miRNAs from the *miR-200* family showed up-regulation in both lymph node and liver metastases. *miR-141* showed a completely different pattern to other *miR-200*, with a significant down-regulation in liver metastases and an insignificant up-regulation in the lymph node metastases. This result is different from previously reported studies. Hur et al. demonstrated a significant up-regulation of *miR-141* in liver metastases (24), whereas no significant change between the two sites was observed by Cristobal et al. (35).

Interestingly, we observed some differences between lymph node and liver metastases. Significant up-regulation in both lymph node and liver metastases was observed for *miR-200a*, *miR-200c* and *miR-429*, whereas *miR-200b* was significantly up-regulated only in lymph node metastases. Furthermore, up-regulation was more pronounced in lymph node metastases than in liver metastases, which was an unexpected finding but is in accordance with a recent research suggesting that lymph node and distant metastases develop through fundamentally different evolutionary mechanisms (36).

We also investigated the expressions of genes *PTPN13, ONECUT2, TGFB2, ZEB2, SOX2, RND3* and *CDKN1B*, previously validated as target genes of the *miR-200* family in CRC (37). Among these genes, we focused our attention on those with inverse expression to their miRNA partners, either at the invasive front or in metastases.

At the invasive tumor front, *TGFB2* was the only gene with inverse expression to the *miR-200* family. This is in accordance with our previous study demonstrating a negative correlation between *TGFB2* and three of five members of the *miR-200 family*, namely *miR-200a*, *miR-200c* and *miR-141*, in CRC with lymph node metastases (19). Increased expression of *TGFB2* mRNAs and protein has also been reported in CRC progression (38, 39).

In the metastases, we observed inverse expression of *ONECUT2* and *SOX2* in comparison to the *miR-200* family. Interestingly, *ONECUT2*, a target of *miR-429*, was down-regulated only in lymph node but not in liver metastases. An emerging role of *ONECUT2* in tumor biology has recently been recognized (40, 41). It has been shown that *miR-429* inhibits the initiation of EMT by targeting *ONECUT2* (42). It has also been suggested that *miR-429* could reverse TGF-β induced EMT by interfering with *ONECUT2* in CRC (42). However, neither *miR-429* nor *ONECUT2* expression have been previously described in CRC metastases. Our results suggest that *miR-429* may contribute to lymph node metastasis formation by reversing EMT related changes in CRC. *SOX2*, a target of both *miR-200c* and *miR-429*, is a stemness transcription factor, promoting proliferation, migration and invasion in CRC (43, 44). We observed down-regulation of *SOX2* in both lymph node and liver metastases in comparison to the invasive front. It has been previously suggested that reduced expression of *SOX2* causes the restoration of growth and metastasis *in vivo* and *in vitro* (43). Expression of *SOX2* has been previously investigated in CRC liver metastases (45, 46). However, there is limited data about the expression of *SOX2* in lymph node metastases, and on comparison between its expression in metastases and primary CRC. Our results suggest that down-regulation of *SOX2* in distant metastases may play a role in metastases formation. This is in accordance with previous research showing that down-regulation of *SOX2* by *miR-429* decreases cell apoptosis (44).

Furthermore, we were interested in genes that showed inverse expression in metastases compared to the invasive tumor front, irrespective of their regulatory miRNAs expression. We found *CDKN1B*, *PTPN13* and *ZEB2* to be down-regulated at the invasive front and up-regulated in the metastases. *CDKN1B* is a target of *miR-200b*, which is believed to have a tumor-promoting role in CRC (47). A previous study showed that in CRC, loss of p27 protein, encoded by *CDKN1B*, probably promotes lymph node metastases and is correlated with a poor prognosis (48). Prior research on an mRNA level found only discrete changes in protein expression between liver metastases and the primary tumor (48). In contrast, our findings showed *CDKN1B* gene down-regulation in lymph node metastases, suggesting an important role of post-transcriptional regulators, such as miRNAs. While there are no available data about the expression of *PTPN13* (*miR-200c* target) in metastases, a number of its potential interacting partners point to its role in tumor progression, including modification of cell shape, motility and cell signaling (49, 50). *PTPN13* has been reported to be an anti-apoptotic factor in CRC (51). There is also little information about the *RND3* gene. In our study, it showed only a slight increase in expression between the invasive front and lymph node and liver metastases. This result is consistent with a previously described elevated expression of the RND3 protein in lymph node metastases compared to the primary tumor (52). *ZEB2*, a target of all *microRNAs* of the *miR-200* family, is an EMT-inducing transcription factor, previously shown to be associated with progression of CRC and a risk of distal but not local recurrence (53, 54). It has been shown that it is overexpressed at the invasion front of liver metastases in comparison to the center of metastases (55).

There are several advantages and limitations of our study. The main advantage is the use of the micropuncture technique, which enabled us to obtain tissue from the locations of interest, determined by microscopic analysis. Importantly, direct comparison of distinct locations within the tumor and matched metastases from the same patient enabled the avoidance of inappropriate comparison of miRNA expression between different tumors/patients. This is important, since miRNAs might be tissue specific, and depend on age and gender (56). However, using this technique, both tumor and stromal cells were present in tissue samples, not allowing a comparison between the tumor and stromal cells. Another limitation of our study is a small number of patients with heterogenous tumors regarding the stage, tumor budding and poorly differentiated clusters. Correlation between these features and the expression of EMT markers was therefore not possible. Furthermore, patients’ selection was biased because only those with lymph node and/liver metastases were included. The study groups were also uneven, since a sufficient number of patients matching the designed groups could not be obtained.

**Figure 5:**
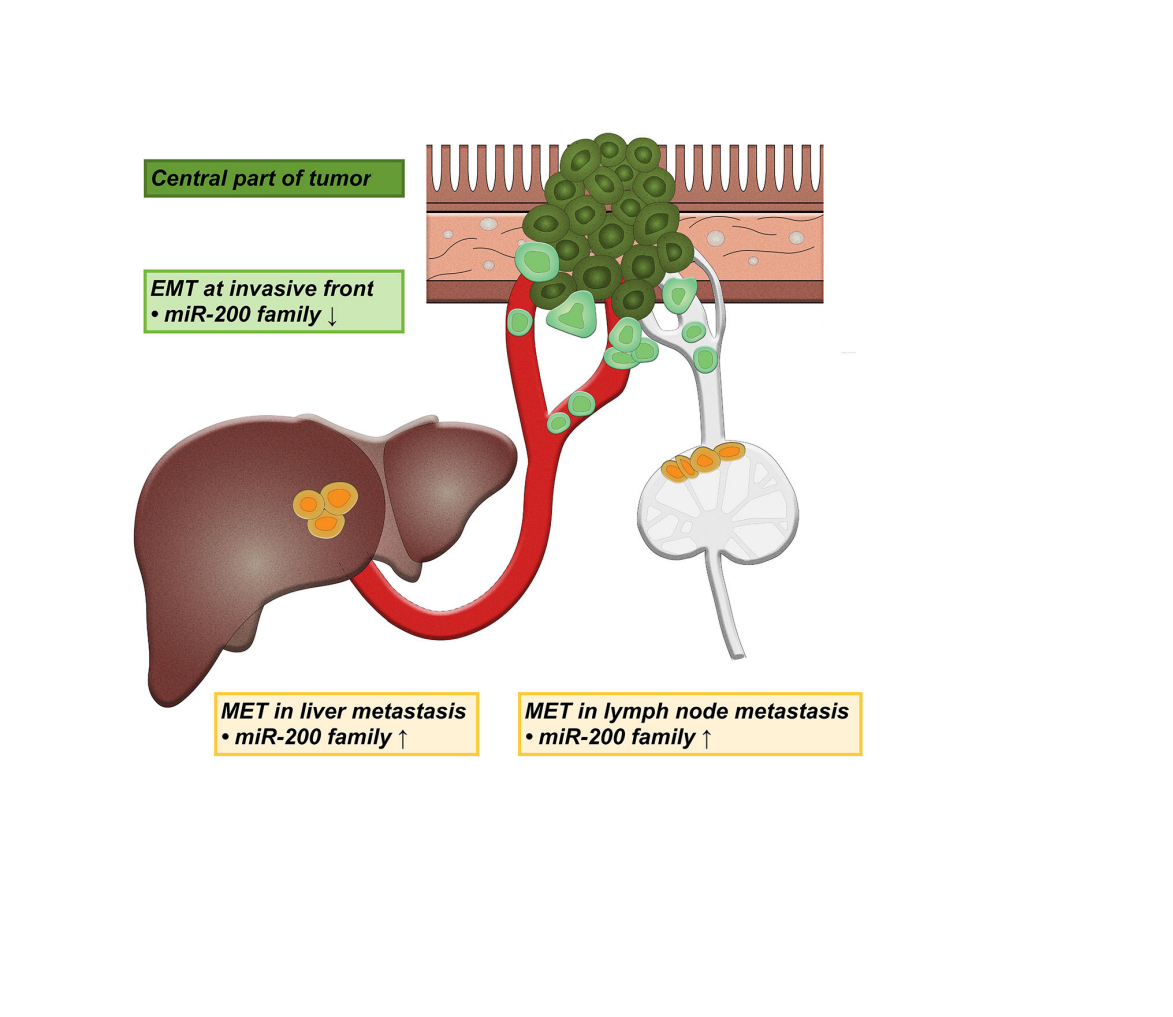
Schematic representation of the involvement of partial epithelial-mesenchymal transition (EMT) and partial mesenchymal-epithelial transition (MET) in lymph node and liver metastases. ↑ up-regulation, ↓ down-regulation.

In conclusion, our results suggest the involvement of partial EMT at the invasive tumor front, and a reverse process - partial MET in both lymph node and liver metastases in CRC, based on analysis of the *miR-200* family expression (**Figure 5**). The expression patterns of some of the postulated target genes further support these results. Interestingly, some differences were observed between lymph node and liver metastases, encouraging further studies, which will hopefully result in the development of new treatment modalities.

## Data Availability Statement

The raw data supporting the conclusions of this article will be made available by the authors, without undue reservation.

## Ethics Statement

Patient consent was waived due to the following reason. As stated in the approval document, the study is retrospective, observational, performed on tissue samples that were obtained during routine diagnostic/therapeutic procedures, consisted of either excision or resection. Therefore, enough tissue was available for routine analysis and research. Moreover, tissue is still available for any additional analysis in the future. Our State Ethical Committee does not require informed consent from patients in such studies. However, the informed consent was obtained before the routine procedure.

## Author Contributions

Conceptualization, NZ, AP and EB. Methodology, AP, KU, KŠ, EB and NZ. Software, KU and EB; validation, AP, KU and EB. Formal analysis, AP, KU, KŠ, EB and NZ. Investigation, AP, KŠ, KU and EB. Resources, NZ. Data curation, AP, KU, KŠ, EB and NZ. Writing—original draft preparation, AP. Writing—review and editing, AP, KU, EB and NZ. Visualization, AP and NZ. Supervision, EB and NZ. All authors contributed to the article and approved the submitted version.

## Funding

This work was supported by the Slovenian Research Agency (ARRS) under research core funding number P3-0054.

## Conflict of Interest

The authors declare that the research was conducted in the absence of any commercial or financial relationships that could be construed as a potential conflict of interest.
